# Argon used as dry suit insulation gas for cold-water diving

**DOI:** 10.1186/2046-7648-2-17

**Published:** 2013-06-03

**Authors:** Xavier CE Vrijdag, Pieter-Jan AM van Ooij, Robert A van Hulst

**Affiliations:** 1 Department of Hyperbaric Medicine, Academic Medical Center, University of Amsterdam, Amsterdam 1105 AZ, The Netherlands; 2 Diving Medical Center, Royal Netherlands Navy, Den Helder 1780 CA, The Netherlands; 3 Laboratory of Experimental Intensive Care and Anesthesiology (L.E.I.C.A.), Department of Anaesthesiology, Academic Medical Center, University of Amsterdam, Amsterdam 1105 AZ, The Netherlands

**Keywords:** Diving, Cold effects, Argon, Dry suit, Inflation gas, Hypothermia, Thermal insulation

## Abstract

**Background:**

Cold-water diving requires good thermal insulation because hypothermia is a serious risk. Water conducts heat more efficiently compared to air. To stay warm during a dive, the choice of thermal protection should be based on physical activity, the temperature of the water, and the duration of exposure. A dry suit, a diving suit filled with gas, is the most common diving suit in cold water. Air is the traditional dry suit inflation gas, whereas the thermal conductivity of argon is approximately 32% lower compared to that of air. This study evaluates the benefits of argon, compared to air, as a thermal insulation gas for a dry suit during a 1-h cold-water dive by divers of the Royal Netherlands Navy.

**Methods:**

Seven male Special Forces divers made (in total) 19 dives in a diving basin with water at 13°C at a depth of 3 m for 1 h in upright position. A rubber dry suit and woollen undergarment were used with either argon (*n* = 13) or air (*n* = 6) (blinded to the divers) as suit inflation gas. Core temperature was measured with a radio pill during the dive. Before, halfway, and after the dive, subjective thermal comfort was recorded using a thermal comfort score.

**Results:**

No diver had to abort the test due to cold. No differences in core temperature and thermal comfort score were found between the two groups. Core temperature remained unchanged during the dives. Thermal comfort score showed a significant decrease in both groups after a 60-min dive compared to baseline.

**Conclusions:**

In these tests the combination of the dry suit and undergarment was sufficient to maintain core temperature and thermal comfort for a dive of 1 h in water at 13°C. The use of argon as a suit inflation gas had no added value for thermal insulation compared to air for these dives.

## Background

Cold-water diving is a situation that requires good thermal insulation because hypothermia is a serious risk. A core temperature of 36°C creates a loss of mental performance, and the diver may experience difficulties in accomplishing operational tasks. With a further decrease of 0.5°C, the diver can barely carry out basic functions needed to maintain dive safety [1]. Professional divers and military divers working in the waters of northwest Europe often make dives in the order of 2 to 3 h before surfacing. Also, a small group of the sport diving community (called technical divers) performs long dives to depths of ≥40 m in waters with temperatures down to 3°C.

Water has a higher thermal conductivity and heat capacity than air. In addition, water movement around the diver causes convection which results in a further increase in heat loss. In order to maintain thermal homeostasis during a dive, thermal protection needs to be chosen based on physical activity, the temperature of the water and the duration of exposure [2].

Below 10°C of water temperature, a wet suit will not keep the diver warm when exposed for 30 min or longer [3,4]. Dry suits are advised for water temperatures ≤21°C. An undergarment beneath a dry suit relies on entrapment of gas (usually air) in the interstitial spaces of the garment material for thermal insulation [5].

Air has been the most convenient and frequently used dry suit inflation gas, with some exceptions. Helium is a component of the breathing gases (trimix) for deeper dives [6]. Thermal insulation was found to decrease by 71% with the use of trimix for dry suit inflation compared to air as insulation gas [7]. Therefore, other gases have been studied for the use of dry suit inflation. When argon was compared with 13 other gases, it appeared to be a good option based on its physical properties, i.e., it is monoatomic and has a large size and high mass, causing low thermal conductivity [8]. The thermal conductivity of argon is about 32% lower compared to that of air [8]. Other inert gases, like krypton and xenon, are also (theoretically) possible, but their high costs make them impractical for diving operations.

Within the technical diving population, argon is regularly used as dry suit inflation gas for deeper dives with helium-based breathing gas mixtures [9]. In a study with thermal manikins, a significant improvement in dry suit thermal protection was found when using argon [8]. However, in a study with human divers, no effect was found when measuring thermal comfort and core and skin temperature [10].

This disagreement, in conclusion, resulted in the present study evaluating the benefits of argon, compared to air, as a thermal insulation gas for dry suits during cold-water diving.

## Methods

### Subjects

Seven male Special Forces divers from the Royal Netherlands Marine Corps volunteered to participate in this study. All were medically fit [11] and certified ‘fit to dive’. They were experienced oxygen rebreather divers. This study was approved by the Surgeon General and the Superintendent of Diving (Royal Netherlands Navy). During this study, no invasive procedures or blood tests were performed, and all dives were standard operational dives for the Special Forces divers; therefore, an institutional review board (IRB) approval was not questioned in advance of the experiments by the research group.

Information about the aim of the study was given, and risks were explained. All participants were told that they could withdraw from the study at any time and that the results would not be archived in their medical file. Any questions concerning the research could be discussed with an independent physician. Before the dives, all divers were asked again if they had any questions or objections regarding the study. When there was none, they all signed a written informed consent. The local ethics committee determined that IRB approval was not necessary for the current analysis because the measurements were initially completed as part of operational procedures and was only later considered for scientific publication.

### Diving procedure

The dives were made in the diving basin of the Royal Netherlands Navy at a depth of 3 m for 60 min or shorter if a diver indicated that he was feeling uncomfortable or cold. The normal procedure for diving using a LAR VII oxygen rebreather (Draeger, Lübeck, Germany) was followed. Divers using this equipment are breathing 100% oxygen. During the study, the divers were sitting still in upright position on a bench with moderate negative buoyancy. Each diver made three dives: one dive with air as suit inflation gas and two dives with argon as suit inflation gas. For thermal considerations, each diver made only one dive each day. The water in the basin was refreshed every day to keep the water at a constant temperature. During all measurements, the water temperature was 13.0°C ± 0.5°C.

### Dry suit inflation gas

A navy dive master, not involved in this study, filled the bottles used for dry suit inflation. The bottles were filled with either compressed air or with 100% argon. The compressed air was made with a compressor at the naval base and complies with EN 12021 ‘Respiratory protective devices - compressed air for breathing apparatus’ [12]. Argon (Linde Gas, Schiedam, The Netherlands) was delivered in compressed buffers (Gas certificate ensuring <0.004% impurities, balance Ar) which were used to fill the dry suit bottles. All bottles were numbered, and the numbers registered and stored for later analysis. All divers and the diver supervisors were blinded from the type of insulation gas used. The order of bottle usage was random. In this study, the term ‘argon divers’ or ‘air divers’ refers to the type of inflation gas, not to the breathing gas, used.

### Flushing dry suit procedure

A Viking Pro1000 rubber dry suit (Trelleborg Protective Products AB, Trelleborg, Sweden) and woollen undergarment were used. For all dives, the same combination of dry suit and undergarment was utilized. Flushing of the dry suit was done as described by Risberg and Hope [10]. Briefly after the pre-dive procedures, the dry suit was deflated as much as possible and completely filled with the inflation gas. When entering the water, the dry suit was completely deflated and maximally inflated again. This procedure was repeated three times. Hereafter, the diver descended to the floor of the basin and inflated the dry suit according to the diver's personal preference. This ensured that the dry suit is completely filled with the inflation gas.

### Temperature measurements

To measure the core temperature of the diver, CorTemp® was used; this is an ingestible core body temperature sensor used in conjunction with a CorTemp® data recorder (HQ Inc., Palmetto, FL, USA). Communication between the data recorder and sensor is via radio waves. The recorder was calibrated according to the information provided by the manufacturer. In accordance with the manufacturer's guideline, the temperature sensor was swallowed by the diver 1 h before the dive. The recorder was worn under the dry suit, on the skin of the diver, to enhance signal transduction. The thermometer pill is harmless to the body and is defecated after 24 to 72 h. The sensor has an accuracy of ±0.1°C and is designed for human use.

In addition to the core temperature measurement, the divers were asked before, halfway, and at the end of the dive to report on their subjective impression of thermal comfort using an ordered categorical 0 to 10 scale (where 0 represents ‘most uncomfortable’ and 10 represents ‘most comfortable’).

### Statistical analysis

Testing for normality was done with the Kolmogorov-Smirnov test. Core temperature meets normality criteria, whereas the thermal comfort score is not normally distributed. Between-group differences (air versus argon) in mean core temperature over time were analyzed with a general linear model. An independent *t* test was used to evaluate differences in core temperature between the air and argon groups at 0 min. To test for differences in core temperature between 0 and 60 min for each group, paired *t* tests were used. The Mann-Whitney test was used to evaluate differences, grouped by type of gas, in the thermal comfort score between the two groups. The Wilcoxon signed-rank test was used to evaluate differences in the thermal comfort score between 0 and 60 min. A *p* value <0.05 was considered statistically significant. Analyses were done using SPSS statistics version 19 (IBM Inc, Armonk, NY, USA).

## Results

Data on the seven male divers are presented in Table 1. No problems (e.g., a flooded dry suit) occurred during any of the dives. None of the divers terminated the dive earlier than 1 h because of feeling cold or being uncomfortable. Two divers were unable to make one dive in the series of three dives: one diver's case was due to operational deployment; the other, because of an ear problem. Therefore, the results on thermal comfort are based on 6 air and 13 argon dives. Core temperature data points of two dives had to be excluded because of extreme outliers (i.e., ≥80°C and ≤15°C). Finally, analysis of the core temperature was performed on 5 air dives and 12 argon dives.

**Table 1:** Subject characteristics

**Characteristic**	**Mean**	**Standard deviation**
**Age (years)**	32	8
**Weight (kg)**	87	4
**Height (cm)**	183	6
**Fat (%)**	14.3	8.0

### Core temperature

In all dives, the core temperature decreased at 60 min compared to 0 min. There were no statistically significant differences in core temperature between the two groups. However, there was a significant decrease in core temperature between 0 and 60 min in those dives using argon, in contrast to the dives using air as inflation gas, where no difference was found (Table 2).

**Table 2:** Core temperature during a 1-h dive

**Insulation gas**	**0 min**	**60 min**
Air (n = 6)	37.3 (0.5)	37.3 (0.4)
Argon (n = 13)	37.6 (0.3)	37.4 * (0.2)

Mean core temperature (standard deviation in parentheses). *p < 0.05; significant difference compared to 0 min.

**Figure 1:**
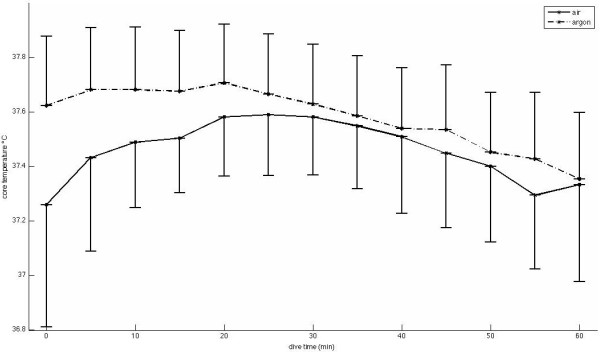
**Core temperature.** Core temperature while using air (solid line; n = 5) or argon (dashed line; n = 12) as inflation gas in the dry suit for a 1-h dive. Shown are mean values and standard deviations.

Mean core temperature measurements increased during the first 20 to 30 min (Figure 1), but the change from control measurements was not statistically significant. Thereafter, core temperature began to decrease. Differences between the maximum and minimum temperature during a dive were (on average) 0.5°C for all dives (air and argon), with a maximum difference of 1.3°C and a minimum difference of 0.2°C. One diver in the air group started with a lower core body temperature of 36.5°C compared to the other dives, resulting in larger standard deviations in the (small) air group. The lowest core temperature in the argon group was 37.0°C; in the air group, this was the starting temperature mentioned above.

### Thermal comfort

At the start of the dives, for both the argon and air dives, the divers felt comfortable with a median thermal comfort score of 8. After 30 min there was no difference in the thermal comfort score, whereas after 60 min there was a significant decrease in both groups (Table 3).

**Table 3:** Subjective thermal comfort during a 1-h dive

**Insulation gas**	**0 min**	**30 min**	**60 min**
Air (*n* = 6)	8 (6 to 8)	6.5 (5 to 7)	6* (5 to 6)
Argon (*n* = 13)	8 (7 to 9)	7 (6 to 7)	6* (5 to 7)

Median thermal comfort score (range in parentheses). *p < 0.05; significant difference compared to 0 min.

## Discussion

In this study, no significant difference in core temperature and/or thermal comfort were observed comparing argon or air as dry suit inflation gas while diving for 1 h at a water temperature of 13°C.

These results are similar to those reported by Risberg and Hope [10], but the insulation of their divers was different compared to our study. In the study of Risberg and Hope, the divers wore 6-mm foam dry suits so that a large proportion of the total insulation came from the foam. The addition of argon would not change the intrinsic insulation of the foam, and (at a depth of 10 m) the foam makes a major contribution to the total suit insulation. Furthermore, because their divers were in horizontal position, only a small layer of argon was at the front and a larger volume was at the back; this probably led to a greater cooling effect on the chest [10].

In the present study, we used shell dry suits, similar to the manikins used in the study of Nuckols et al. [8], with the divers in upright position. However, using a shell dry suit and a different position in our study did not result in significant differences between argon and air during the dive time of 1 h.

In both our groups, there was an initial rise of core temperature before it began to decrease, although the difference was not statistically significant from the control measurement. This might be due to vasoconstriction of vessels in the skin causing a thicker insulation layer around the body core. Immersion of the skin in cold water elicits an increased translocation of blood to the chest due to vasoconstriction in the skin of the limbs, subcutaneous tissues, and muscles [13,14]. This effect causes a decrease in the ability of the body to lose heat by peripheral cooling. At the beginning of the dive, the insulation of the subcutis, the dry suit and the undergarment make loss of body heat less likely to occur. An alternative hypothesis would be metabolic heat production due to muscular activity while wearing thick undergarment and dry suit. This metabolic heat production may continue for some time even after the diver has been positioned in relative rest in the water. In this situation, there may be a short-lasting temperature increase as heat production exceeds heat loss, through the vasoconstricted skin. If heat loss within the insulation layers exceeds heat production, this will lead to peripheral cooling and eventually to a reduction in core temperature. This might explain the initial (nonsignificant) increase in core temperature before it decreases, as also reported by others [15,16].

In the study of Arieli et al., for dives of 3 h at 17°C to 18.5°C in wet suits, a correlation between core temperature and subjective scoring of the temperature was found at 60 min of diving, but not after that time. They concluded that when divers' core temperature decreases, they are not able to report reliably the extent to which their thermal condition has deteriorated [17]. In our conditions, the core temperature did not drop as far as in the study of Arieli et al. [17]; thus, the thermal comfort score as reported can be used as a reliable measurement for thermal condition.

The situation tested in the present study is relevant, coming from a military operational perspective. Military Special Forces divers use diver propulsion vehicles (DPV) for transport underwater to the operational task; this is a physically passive activity. These divers are sitting on the DPV, and an increased heat flux would be expected from the gluteal area directly in contact with the DPV with a minimal insulation layer. The DPV thrust also causes an increased water flow. As discussed earlier, an increased heat flux can be expected by the flowing water. These divers must be fit and not hypothermic after the dive in order to successfully perform the military task.

In addition, the increasing number of ‘technical divers’ who often dive for 2 to 3 h with long decompression stops when they perform only slight physical activity. During these decompression stops, the divers hover in horizontal position causing a minimal insulation layer on the thoracic and abdominal areas. A decrease in core temperature represents a serious risk for decompression sickness. Being warm during the dive elicits a higher uptake of inert gas in the tissue [18,19], whereas being cold during the decompression phase of the dive decreases the off-gassing efficiency and increases the risk of decompression sickness [19,20]. Therefore, for specific diving populations, more studies are needed on thermal protection in both stagnant and flowing water.

### Limitations

In the present study, the dive time was only 1 h, and with the selected insulation of the dry suit and undergarment, no clinically relevant decrease in core temperature was seen. However, a longer dive time might elicit a greater decrease in temperature, which could make a difference when either argon or air is used as dry suit inflation gas.

Also, no additional factors (e.g., the effect of flowing water) were taken into account. Increased heat flux from the diver to the surrounding water would be expected in normal diving when the diver is surrounded by flowing water compared to the present experimental situation with still water. This increased heat flux might be necessary to obtain an increasing difference between both groups.

Thermal heat loss was measured at the core of the body. However, this measurement will not be sensitive for moderate cold exposure because only with a significant heat loss will the core temperature eventually decrease. Using skin thermistors and core temperature measurements, an indirect assessment of heat loss can be made. To investigate the thermal insulation itself, heat flux sensors can be used. In future research, additional measurement methods might be more useful for the moderate cold stress induced during normal dives.

## Conclusions

The combination of a rubber dry suit and wool undergarment is sufficient for dives of 1 h in water at a temperature of 13°C. For this kind of dive, the use of argon as a suit inflation gas has no additional value for thermal insulation as compared to air. Within the limitations of the present study, we found no positive effects on insulation using argon as inflation gas. Future studies are required, which might come to different conclusions under extreme diving conditions, such as longer dive times, colder water, and/or dives in nonstagnant water.

## Abbreviations

DPV: Diver propulsion vehicle; IRB: Institutional review board.

## Competing interests

The authors declare that they have no competing interests.

## Authors’ contributions

XV did the data analysis and drafted the manuscript. PO did the study design, acquired the data and helped to draft the manuscript. RH conceived the study and participated in its design and coordination, and helped to draft the manuscript. All authors have read and approved the final manuscript.
